# ATP Induces NO Production in Hippocampal Neurons by P2X_7_ Receptor Activation Independent of Glutamate Signaling

**DOI:** 10.1371/journal.pone.0057626

**Published:** 2013-03-05

**Authors:** Juan Francisco Codocedo, Juan Alejandro Godoy, Maria Ines Poblete, Nibaldo C. Inestrosa, Juan Pablo Huidobro-Toro

**Affiliations:** 1 Centro de Envejecimiento y Regeneración, Departamento de Biología Celular y Molecular, Facultad de Ciencias Biológicas, Pontificia Universidad Católica de Chile, Santiago, Chile; 2 Departamento de Fisiología, Facultad de Ciencias Biológicas, Pontificia Universidad Católica de Chile, Santiago, Chile; Albany Medical College, United States of America

## Abstract

To assess the putative role of adenosine triphosphate (ATP) upon nitric oxide (NO) production in the hippocampus, we used as a model both rat hippocampal slices and isolated hippocampal neurons in culture, lacking glial cells. In hippocampal slices, additions of exogenous ATP or 2′(3′)-O-(4-Benzoylbenzoyl) ATP (Bz-ATP) elicited concentration-dependent NO production, which increased linearly within the first 15 min and plateaued thereafter; agonist EC_50_ values were 50 and 15 µM, respectively. The NO increase evoked by ATP was antagonized in a concentration-dependent manner by Coomassie brilliant blue G (BBG) or by N^ω^-propyl-L-arginine, suggesting the involvement of P2X_7_Rs and neuronal NOS, respectively. The ATP induced NO production was independent of N-methyl-D-aspartic acid (NMDA) receptor activity as effects were not alleviated by DL-2-Amino-5-phosphonopentanoic acid (APV), but antagonized by BBG. In sum, exogenous ATP elicited NO production in hippocampal neurons independently of NMDA receptor activity.

## Introduction

Nitric oxide (NO) and ATP are ubiquitous extracellular messengers involved in multiple physiological and pathophysiological processes in the nervous system and peripheral tissues [1]. NO is produced by L-arginine oxidation, a reaction catalyzed by the nitric oxide synthase (NOS) family. nNOS and eNOS are the constitutive enzyme isoforms found predominantly in neurons and endothelial cells, respectively; these two proteins are controlled by Ca^+2^-calmodulin (CaM) in a concentration-dependent manner. In contrast, iNOS is the transcriptionally induced enzyme variant that is up-regulated during inflammation and generates the largest amount of NO. iNOS catalytic activity is independent of intracellular Ca^+2^ oscillations [2]. The NO signaling pathway involves activation of soluble guanylyl cyclase, which subsequently increases intracellular levels of 3′, 5′-cyclic guanosine monophosphate (cGMP). NO may also react with cysteine thiols to S-nitrosylate [3] relevant synaptic proteins such as, stargarzin [4] or the NR2A subunit of the NMDA glutamate receptor [5], among others.

Since the concept of purinergic neurotransmission was coined by Burnstock [6], many actions of ATP have been described in the nervous system at the cellular and molecular level. ATP and related nucleotides are stored in synaptic vesicles, acting either as a sole transmitter or as a co-transmitter together with GABA [7], glutamate [8], acetylcholine, or noradrenaline [9], [10]. Extracellularly released ATP acts on ionotropic receptors (P2XRs) or metabotropic receptors (P2YRs), and both classes of purinoceptors are widely distributed in the brain nuclei and peripheral tissues [11]. Seven P2XRs subtypes have been described; these channels permeable to monovalent cations and Ca^+2^, assemble as homo- or heterotrimeric oligomers. These receptors modulate several signaling pathways including neurotransmitter release from pre-synaptic sites [12].

Although the mechanisms regulating the production and release of NO and ATP are very different, several studies suggest that P2XR activation leads to an increased NO production, proposing a functional link between ATP and NO production. Current data supporting this hypothesis, are mainly derived from studies on astrocytes [13]–[15], however scarce evidence is available to support this regulatory mechanism in neurons. To date, no experimental studies have focused on demonstrating the influence of ATP, or related nucleotides, on the production and release of NO by hippocampal neurons. Moreover, the putative nucleotide receptors that might be involved in this pathway have been identified. Given that ATP and its receptors are involved in brain excitability and in long-term potentiation (LTP) [16], studies of this functional link are highly relevant, particularly in the hippocampus. Trace metals such as 1–10 µM zinc have been shown to increase the magnitude of the ATP component of hippocampal LTP [16]. Therefore, we deemed relevant to ascertain whether nucleotides and purinoceptors are involved as regulators of brain excitability. In this communication, we demonstrate, for the first time, that in rat hippocampus slices, as in isolated hippocampus neurons, the application of exogenous ATP increases NO production through P2X_7_R activation. Since the increase in NO production is resistant to APV, a classical NMDARs antagonist, we conclude that this effect is independent of glutamate NMDARs activation.

## Materials and Methods

### Ethics Statement

Sprague-Dawley rats were housed in the University Animal Facility and handled according to guidelines outlined and approved by the Institutional Animal Care and Use Committee at the Faculty of Biological Sciences of the P. Universidad Católica de Chile.

### Dissection of Rat Hippocampus; Preparation of Slices

Adult Sprague-Dawley rats (250 g) were anesthetized with xylazine-ketamine and then decapitated. Brains were immediately immersed in an ice-cold dissection buffer of the following composition (in mM): sucrose 110, NaCl 60, NaHCO_3_ 28, NaH_2_PO_4_ 1.25, KCl 3, MgSO_4_ 7, CaCl_2_ 0.5, glucose 5, (bubbled with a gas mixture of 95%O2/5%CO2 to maintain pH levels at 7.2–7.3 ). Both hippocampi were dissected from the whole brain and cut into blocks of 1 mm and maintained for 1 hour in artificial cerebrospinal fluid (ACSF) solution of the following composition (in mM): NaCl 124, NaHCO_3_, 25 NaH_2_PO_4_ 1, KCl 4.4, MgSO_4_ 1.2, CaCl_2_ 2, glucose 10 (bubbled with a gas mixture of 95%O2/5%CO2 to maintain pH levels at 7.3–7.4). Slice preparations and subsequent experimental procedures were conducted at room temperature.

### Culture of Rat Hippocampal Neurons

Primary hippocampal neurons were obtained from 18-day-old Sprague-Dawley rat embryos and maintained in Dulbecco’s modified Eagle’s medium supplemented with 10% horse serum for 2 h. The culture medium was then substituted with Neurobasal medium supplemented with B27, 100 µg/ml streptomycin, and 100 units/ml penicillin. At 3 and 7 days in vitro (DIV) cells were treated with 2 µM araC for 24 h to reduce the number of glial cells present in the culture. At 15 DIV, cultured hippocampal neurons were used for various experiments; cells were maintained and experiments were conducted at 37°C. The percentage of glial contamination was estimated by Glial fibrillary acidic protein (GFAP) immunostaining at 14 DIV. 30 microscopic fields were analyzed per coverslip from two individual cultures.

#### Nucleotide applications; dose-response and time-course studies of NO production

To assess the putative nucleotide receptor(s) involved in NO production, several nucleotides with preferential affinity for the different nucleotide receptors were applied (10–300 µM) to either slices or cultured neurons. Dose–response data points were fitted by a three-parameter logistic equation using a nonlinear curve-fitting program that derives the EC_50_ value of the curves (GraphPad Prism 5.0, San Diego, CA, USA). The equation used for curve fitting was: y = Emax/[1+ (EC50/x)nH ], where y is the magnitude of the NO production (pmol) elicited by ATP (normalized in all cases), Emax is the maximal NO production, EC_50_ is the agonist concentration producing 50% of the maximal effects, nH is the Hill coefficient, and x is the concentration of ATP. Time course studies were performed in hippocampal slices with a battery of ligands including α,β-MethyleneATP (α,β-MeATP), adenosine diphosphate (ADP), 2 methyl thio ADP (2-MeSADP), uridine 5′-diphosphate (UDP), uridine 5′-triphosphate (UTP) or adenosine. Experiments were repeated in a minimum of 3 separate rats. Aliquots of the tissue or cell buffer media (ACSF) were sampled for NO production as a part of dose-response or time course studies.

#### Blockade of nNOS activity

To examine whether the NO production elicited by 100 µM ATP applications to hippocampal slices was due to nNOS activity, we used N^ω^-propyl L-arginine, as a selective inhibitor of this isoenzyme [17]. The slices were incubated with 10–200 nM of this enzyme inhibitor 30 min prior to ligand applications; (n = 3–4 per concentration). As a control, non-treated hippocampal slices were assessed (n = 4). In separate experiments, hippocampal slices from 2–3 rats were incubated with 100 µM N^ω^-nitro-L-arginine, a non-selective NOS blocker.

#### Use of selective receptor antagonists to identify the participation of P2X_7_R or NMDA receptor

Since Bz-ATP evoked a robust NO production, we suspected the possible involvement of the P2X_7_R. Consistently, in separate experiments we assessed whether BBG (1–1000 nM) antagonized the NO production elicited either by 100 µM ATP or 100 µM Bz-ATP applications. Next, we ascertained whether a NMDA antagonist blocked the 100 µM ATP-induced NO surge. To this aim, we used 100 µM ATP in the presence or absence of 10 µM APV (n = 7) or 10 µM Dizocilpine hydrogen maleate (MK-801) (n = 3). We also examined whether 10 µM APV or MK-801, inhibited NO production elicited by 10 µM NMDA (n = 7). As a control for this series of experiments, we tested whether 100 nM BBG (n = 4), 10 µM APV (n = 7) or 100 µM MK-801 (n = 3) alone, with no need for agonist presence, altered basal NO production.

To assess whether NO production occurred through independent pathways, we ascertained whether the co-application of 50 µM ATP plus 1 µM NMDA (n = 3, each) elicited additive effects. Additional experiment were performed using 100 µM ATP plus 10 µM NMDA in the presence or absence of 100 nM BBG or 10 µM APV (n = 3, each).

#### Quantification of NO release by chemiluminiscence

The NO content in aliquot samples from either hippocampal slices or hippocampal culture media, was quantified using a Sievers 280 NO analyzer. Sample measurements were carried out within an hour after aliquot collection. In brief, 50 µl aliquot samples were injected to the reaction chamber of the equipment which was filled with 8 mL of glacial acetic acid containing 100 mg of potassium iodide. This reaction mixture reduced the nitrites contained in the biological samples to NO, since due to the short half-life of NO, this agent was most likely oxidized by the tissue oxygenation. A 50-µL perfusate aliquot was injected into the reaction chamber, and a stream of nitrogen carried the resulting NO to an assay cell, in which the NO-ozone reaction generated chemiluminiscence that was detected by a photomultiplier. Calibration of the equipment was performed routinely with 10–1000 nM sodium nitrite. The sensitivity of this setup allows detecting a threshold of 0.5–1 pmol NO (10–20 pmolml^−1^). Background buffer; readings from the culture media and conditioned media were subtracted to determine the released NO. Results are expressed either as the time course of the luminally accessible NO (pmol/ml), or as the integrated NO recovered above basal values (pmol) or as a percentage over the control values.

### Drug and Antibody Sources

ATP (trisodium salt), ADP (disodium salt), α,β-MeATP (lithium salt), Bz-ATP (triethylammonium salt), UTP (trisodium salt), UDP (trisodium salt), NMDA, APV, MK-801, suramin and BBG were purchased from Sigma Chemicals (St. Louis, MO, USA). All the salts used to prepare the dissection and the ACSF solution were analytically graded and purchased from Merck Chemicals (Darmstadt, Germany).

Rabbit anti-GFAP and mouse anti-β-III-tubulin were purchased from Promega. Secondary antibodies labeled with ^488^Alexa or ^543^Alexa were obtained from Molecular Probes (Carlsbad, CA, USA).

### Statistical Analysis

Results were expressed as the mean ± S.E.M of the values from the number of experiments, indicated in the corresponding assays and figures. Data were evaluated statistically by Student’s t-test or one way Anova with Tukey’s post-hoc test. p values <0.05 were considered statistically significant.

## Results

### ATP-induced NO Production in Hippocampal Rat Slices or Hippocampal Neurons in Culture

**Figure 1:**
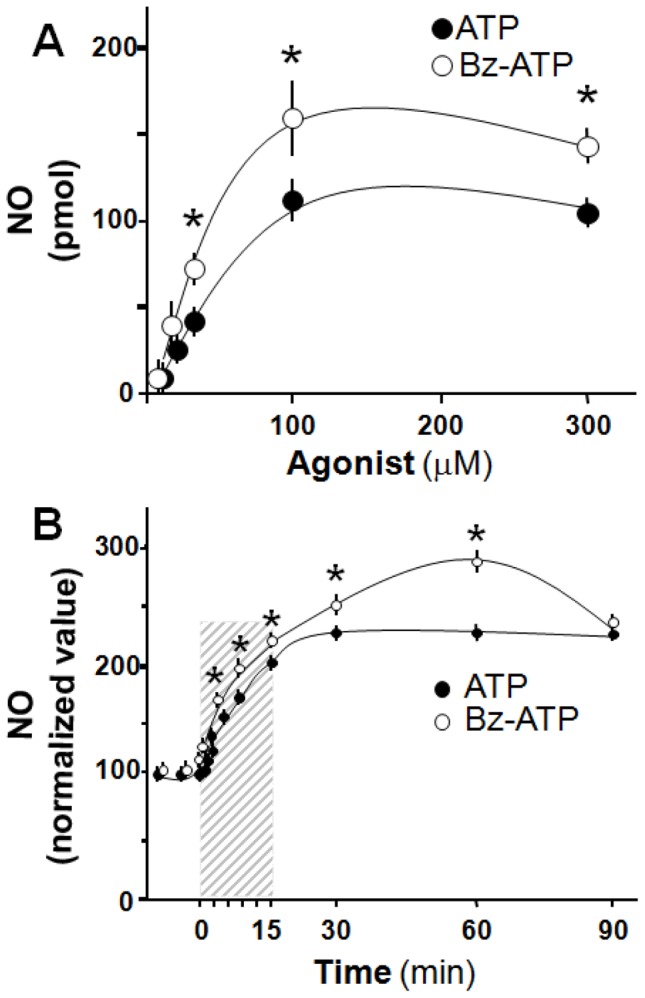
ATP or Bz-ATP induce NO production in hippocampal slices and cultured hippocampal neuron. (**A**): ATP and its analog Bz-ATP, a P2X_7_R preferential agonist, induce NO production in a concentration-depending manner in hippocampal slices; (**B**): In Cultured rat hippocampal neurons (14 DIV), 100 µM ATP or 100 µM Bz-ATP induced a time-dependent surge of NO production, reaching maximal response at 15-min of continuous exposure (shaded bar). In (A) results are expressed as pmol of NO produced per slice. In (B), NO values elicited by nucleotides were normalized with respect to basal values; values represent the mean ± S.E.M, of triplicate experiments, Student’s t-test *p<0.05.

Results compare data obtained from both slices of adult rat hippocampus and cultured hippocampal neurons treated with 1-β-D-arabinofuranosylcytosine (AraC) to eliminate the influence of glial cells. Analysis of GFAP immunostaining established that our cultures contained at most a 6.9±1.0% of glial contamination compared with neurons (Fig. S1). Neuronal cells were stimulated by applying exogenous ATP at 14 DIV, a stage of peak development of synaptogenic process. In both experimental models, ATP evoked a concentration-dependent burst of NO production (Fig. 1A); the maximal increase in NO was achieved with 100 µM ATP. Likewise, in hippocampal cultured neurons, the addition of 100 µM ATP elicited the maximal NO production within 15 min (Fig. 1B).

### Pharmacological Identification of the Possible Nucleotide Receptor and Mechanism Involved in the ATP Mediated-NO Production, Using Hippocampal Slices

**Figure 2:**
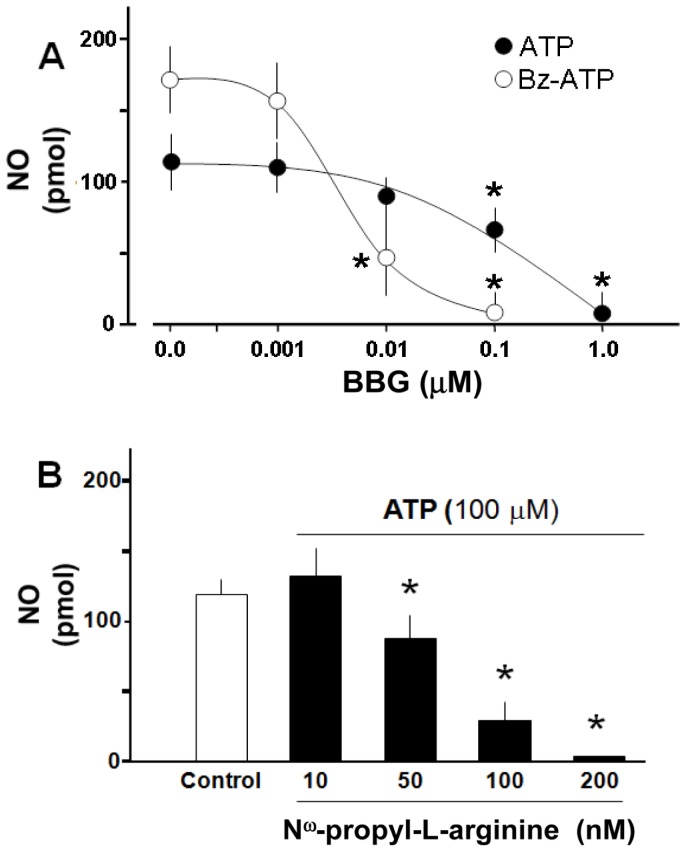
NO production increased by ATP or Bz-ATP involves P2X_7_R and nNOS activation in hippocampal slices. (**A**): BBG, a selective P2X_7_ receptor antagonist, inhibits in a concentration-dependent manner the production of NO mediated by 100 µM ATP or 100 µM Bz-ATP. (**B**): N^ω^-propyl-L-arginine, the selective nNOS inhibitor reduces the NO production elicited by ATP in a concentration-dependent manner. Results represent the mean ± S.E.M, of triplicate experiments, (n = 3–5 separate experiments). Student’s t-test *p<0.05 among nucleotides (A), or as compared to control (B); **p<0.01.

We next used a series of ATP analogues to tentatively identify the putative nature of the purinoceptors involved in the regulation of NO production. Bz-ATP generated an increase in NO production with features similar to those observed with ATP (Fig. 1A). In contrast, neither α,β-methylenATP, 2-MeSADP, ADP, ADO, UTP, nor UDP increased NO production in the slices (Table 1) or in cultured neurons (data not shown). Moreover, pretreatment of hippocampal slices with BBG, a selective P2X_7_R antagonist, reduced in a concentration-dependent manner the production of NO elicited by either 100 µM ATP or 100 µM Bz-ATP (Fig. 2A); the NO production by both agonists was abolished by BBG. Responses induced by Bz-ATP were 10-fold more sensitive to BBG antagonism than ATP. Additionally, we evaluated the NO production mediated by ATP using suramin, a broad-spectrum P2 receptors antagonist [18]. In this experiment, 10 µM suramin decreased basal NO levels (143.4±19.1 vs 73.7±8.7 pmol, n = 4, p<0.05). Moreover, a second application of 100 µM of ATP failed to increase the NO production (73.7±8.7 vs 65.1±7.0 pmol, n = 4, p = 0.19) further supporting the role of purinergic receptors in hippocampal NO production.

**Table 1:** NO production in hippocampal slices induced by purinergic agonists.

Agonist	NO (pmol)
**100 µM ATP (24)**	111.2±12.1***
**100 µM α,β MeATP (3)**	1.5±0.7
**100 µM Bz-ATP (3)**	160.2±38.9***
**100 µM ADP (5)**	1.1±0.5
**100 µM ADO (3)**	0.4±0.3
**1 µM 2-MeADP (3)**	9.4±4.8
**1 µM UTP (3)**	8.4±1.5
**1 µM UDP (3)**	9.8±5.3

Values are means ± S.E.M. No. of slices in parenthesis.

*** vs vehicle (ACSF), p<0.001 Students t-Test.

We next ascertained whether blockade of NOS inhibited the ATP-induced NO synthesis. Incubation of slices with 100 µM N^ω^-nitro-L-arginine, a non selective nNOS inhibitor abrogated the NO production evoked by 100 µM ATP (76.6±12.1, n = 2, vs 16.9±4.3 NO pmol, n = 4). The identity of the enzyme isoform involved in the production of NO was evaluated using N^ω^-propyl-L-arginine, a selective nNOS inhibitor. This blocker reduced dose-dependently the NO rise elicited by 100 µM ATP (Fig. 2B) in concentrations at least 1000-fold lower than N^ω^-nitro-L-arginine.

### The ATP-induced NO Production is Independent of NMDA Receptor Activity

**Figure 3:**
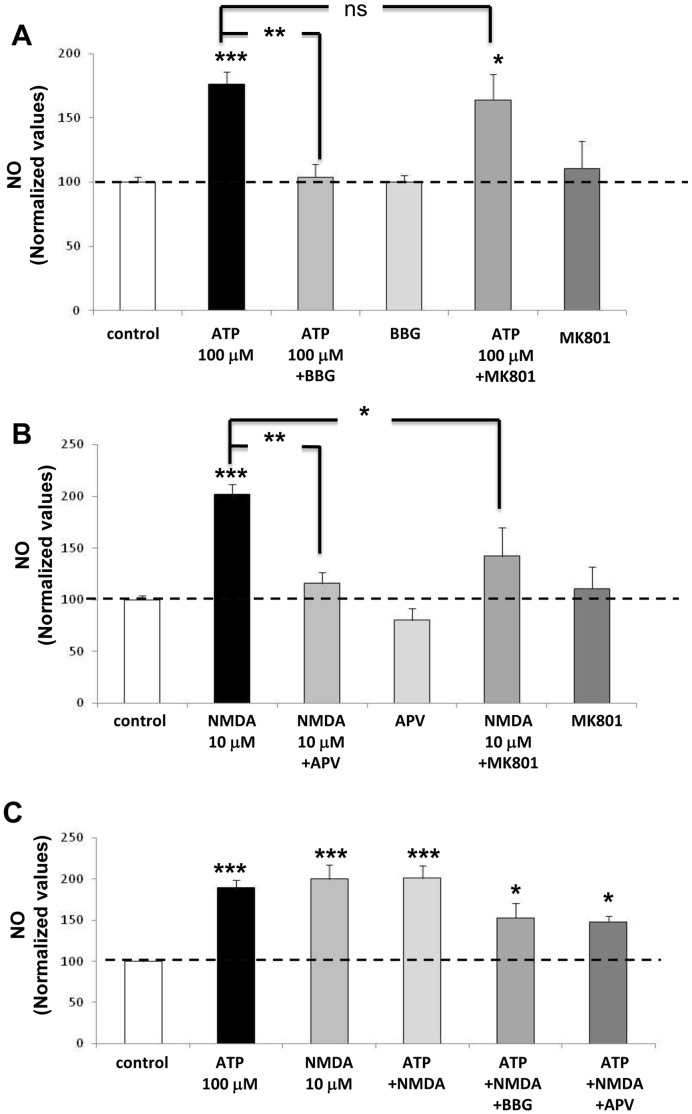
Purinergic and glutamatergic signaling induces NO production via independent mechanisms in cultured hippocampal neurons. (**A**): The 100 µM ATP-induced NO synthesis was abrogated by 100 nM BBG, but not by 10 µM MK-801. (**B**): 10 µM NMDA, used as internal control, induces a NO production that was blocked by 10 µM APV or 10 µM MK-801. In these experiments none of the three antagonists used, affected the basal NO production. (**C**): The co-application of 100 µM ATP plus 10 µM NMDA did not induce a larger NO production than that elicited by each agonist alone. However, the co-application of these ligands plus either 100 nM BBG or 10 µM APV, partially reduced the NO production. These results are shown as normalized NO values (pmol). Columns represent the mean values, bars the S.E.M, of triplicate experiments from 3–4 separate hippocampal cultures. *p<0.05, **p<0.01, ***p<0.001 as compared to the control condition; ns denotes no significant difference (Tukey’s post-hoc test).

To discard the influence of glial cells in the ATP-induced NO production assessed in hippocampal slices, we next examined the action of ATP in cultured hippocampal neurons, devoid of substantial glial contamination. The application of 100 µM ATP elicited a significant increase in NO production; and this effect was inhibited by co-application of 100 nM BBG but not by 10 µM MK-801. Neither BBG alone, nor MK-801, modified basal levels of NO production. The rise of NO elicited by ATP in the presence of MK-801 was not significantly different from control values (Fig. 3A). Moreover, application of 10 µM NMDA generated a significant increase in NO production (Fig. 3B); to test the specificity of this effect, we used two NMDAR antagonists that operate by different mechanisms. APV competitively inhibits the glutamate binding site of NMDAR, and as we expected, 10 µM APV abrogated the NO production elicited by NMDA (Fig. 3B). MK-801, which binds to the NMDAR pore, preventing the flow of cations through the channel, also blocked the NMDA evoked NO production (Fig. 3B). In addition, in separate experiments using hippocampal slices we also observed that 10 µM MK-801 blocked the NMDA evoked NO production (94.2±10.8 vs 39.2±1.7 pmol NO, n = 4, p<0.05), but did not modify the 100 µM ATP-induced NO synthesis (94.2±16.9, pmol NO, n = 4). Parallel experiments were designed to determine the interaction between purinergic and glutamatergic pathways in cultured hippocampal neurons and to examine possible additive interactions. The co-application of 50 µM ATP plus 1 µM NMDA to isolated cultured hippocampal neurons elicited an increase in NO production with an amplitude that was larger than that attained with each agonist alone (Table 2). Notwithstanding, the co-application of 100 µM ATP plus 10 µM NMDA did not generate an additional increase in NO production compared to that obtained with each individual treatment. This finding may indicate that these concentrations caused a maximum calcium influx mediated by both ligands. In these co-application experiments the addition of either 100 nM BBG or 10 µM APV, only partially reduced the NO production elicited by ATP (Fig. 3C).

**Table 2:** NO production in cultured rat hippocampal neurons.

Agonist	NO (normalized values)
**Control**	100.0±2.8
**ATP 50 µM**	132.4±15.6*, ##
**NMDA 1 µM**	137.8±11.0*, #
**ATP 50 µM+NMDA 1 µM**	183.4±5.1***

Normalized values of NO in pmol after 15 mins of continuous agonist exposure (n = 3 each).

* vs control (p<0.05);

*** (p<0.001),

# vs ATP+NMDA (p<0.05);

## (p<0.01), Tukey post-test.

## Discussion

Within the past years, the significance of ATP as a nervous system transmitter has gained increasing recognition. The newly gained knowledge paved the way for future clinical applications of ATP receptor antagonists that may include neuropathic pain, hyperalgesia, neuroinflammation, injury, ischemia, neurodegenerative disorders, epilepsy, among other pathologies [19]. Herein, we focused on the interaction between purinergic signaling and NO production in the rat hippocampus. To this end, we used both hippocampal slices and cultured hippocampal neurons in the absence of glial cells, ruling out the possible role of the latter in any potential interaction. This dual experimental strategy strengthens our conclusions, considering that the results obtained with each approach are complementary.

Several studies have examined the ATP-induced NO production using cultured rat astrocytes; these studies found that ATP induces NO production in glial cells via a calcium-dependent pathway [13] and additionally facilitates NO synthesis induced by interleukin-1β [14] and lipopolysaccharide [15]. A novel contribution of our present study is the finding that in cultured rat hippocampal neurons, ATP elicited NO production through a mechanism(s) that is independent of NMDAR activity, but directly associated to P2X_7_R activation. Present results demonstrate that single applications of 1–100 µM ATP elicited a significant surge of NO production, both in slices of hippocampus or in cultures of excitatory pyramidal hippocampal neurons devoid of companion glial cells. Several arguments concur to indicate that the P2X_7_R is linked to NO production in hippocampal neurons. To identify pharmacologically the nature of the ATP receptor, we used different purinergic ligands for both P2X or P2Y receptor subtypes and suramin, a non-specific P2 receptor antagonist which blocked the ATP evoked NO production. While the metabotropic P2Y receptor agonists examined did not stimulate NO synthesis, 100 µM Bz-ATP consistently generated a concentration-dependent NO surge significantly larger than that elicited by ATP. This finding supports the putative involvement of P2X receptors. Based on literature reports, we are aware that Bz-ATP is a non-preferential P2X_1_, P2X_2_, P2X_3_ and P2X_7_ receptor agonist (the expression of these purinoceptors in adults hippocampus including P2X_4_, was confirmed by western blot analysis, Fig. S2) [20]. While Bz-ATP has a similar potency in P2X_2_ and P2X_3_ receptors, its activity on P2X_1_ and P2X_7_ receptors is substantially greater than ATP [20]. The involvement of P2X_1_R was ruled out because α,β-MeATP, which is a potent and selective P2X_1_ receptor agonist and virtually inactive at the P2X_7_R [21], in our study, did not evoke a significant rise in NO production. Moreover, to confirm the role of P2X_7_ receptor in the ATP-induced NO production, we found that BBG, a non-competitive, highly potent P2X_7_R antagonist, active in the nM range [22], decreased NO production mediated by ATP or Bz-ATP within concentrations compatible with its selectivity for the P2X_7_R subtype. Taken together, the present pharmacological data are consonant with our proposal that P2X_7_Rs play a pivotal role in ATP-induced neuronal NO production. The NO surge is mediated by neuronal NOS activity. On the other hand, the decrease in the basal levels of NO in hippocampal slices observed after suramin applications may imply that the endogenous levels of NO in the hippocampus, could involve additional P2 receptors, probably present in the glia.

A main feature of this communication deals with the hypothesis that ATP induces NO production in the hippocampus through a non-NMDAR-dependent mechanism. To evaluate this proposal and to assess the independence of the ATP-induced NO synthesis from NMDAR activity, we first tested the effects of NMDA, which served as a positive control, and showed that in our assays, NMDA elicited a robust NO production surge, in accordance with previous studies in several brain nuclei [23]–[26]. This rise in NO was abolished by APV and MK-801, demonstrating the pivotal role of glutamate NMDAR in brain NO production. This finding is fully compatible with literature reports on the mechanisms of NMDAR signaling [23], [24]. A possible mechanism that may account for the ATP-induced burst NO involves the activation of pre-synaptic purinoceptors which are known to facilitate glutamate release [27], [28], and thus elicit indirectly nNOS activation through glutamate type NMDARs [29], [30]. To test this hypothesis, we examined whether NMDAR antagonists interfered with the ATP-induced NO production. Consistently, the present results indicate that neither APV nor MK-801 blocked the ATP-induced NO surges mediated by cultured hippocampal neurons, suggesting that ATP- and NMDA-dependent NO production, must occur through separate, independent pathways. This idea was further reinforced by our observation that the co-application of sub-maximal concentrations of exogenous ATP (50 µM) plus NMDA (1 µM) clearly elicited an additive effect. When we carried out the co-application experiments, with 100 µM ATP plus 10 µM NMDA, which independently elicited maximum NO production, we did not observe an additive effect. Interestingly, under these conditions we observed that either BBG or APV (Fig. 3C), failed to achieve baseline NO values as in Figure 3A and 3B. Since the production of NO is resistant to inhibitors (BBG or APV), it suggests the independent contribution of purinergic and glutamatergic mechanisms to regulation of NO production. However, this resistance to the antagonist action is slightly lower than the production of each agonist alone. Although this difference is not significant, this tendency can have different interpretations. One possibility is that a fraction of the exogenous NMDA mediated production was caused by endogenous release of ATP. Therefore, in the experiment of co-application, BBG would be inhibiting the production mediated by exogenous and endogenous ATP. Altogether, the present data allow to propose that glutamatergic and purinergic pathways are involved in neuronal NO production; however, each activates independent mechanism(s).

The cellular events linking P2X_7_R channel activity and neuronal NO production remain to be elucidated, but they likely rely on the entrance of extracellular calcium, required for nNOS activity. The physiological implications of the present findings may be related to the observation that in different pathological states, co-activation of both pathways might occur [31]–[33]. Interestingly, ATP and NO are proposed to participate in survival process [34], [35], thus it is possible to propose that the interaction of both pathways are a natural response to cell injury. On the other hand, the hippocampus is a tissue involved in memory processing. Both ATP and NO have been reported as modulators of plasticity in long term potentiation [16], [36], a long-lasting increase in synaptic efficacy, considered the molecular basis for learning and memory. Thus, we suggest that the mechanism of NO production elicited by P2X_7_R activation may be involved in regulation of neuronal excitability and play a role in the hippocampus memory processing.

## Supporting Information

Figure S1 **Representative image of glial cell contamination at 14 DIV.** The percentage of glial contamination was obtained by counting the number of GFAP positive cells (green). Neurons are detected by b-III-tubulin immunostaining (red).
(TIF)

Figure S2 **Western blot analysis of P2XR subtypes in hippocampus of adults rats.** The fig. show the detection of P2X1, 2, 4 and 7 receptors, in three different rats. Bars indicate molecular weights in KD.
(TIF)
