# Supplementary figures and images for: ATP Induces NO Production in Hippocampal Neurons by P2X7 Receptor Activation Independent of Glutamate Signaling
Source: PLoS One. 2013 Mar 5;8(3):e57626. doi: 10.1371/journal.pone.0057626 (PMC3589399; doi:10.1371/journal.pone.0057626)

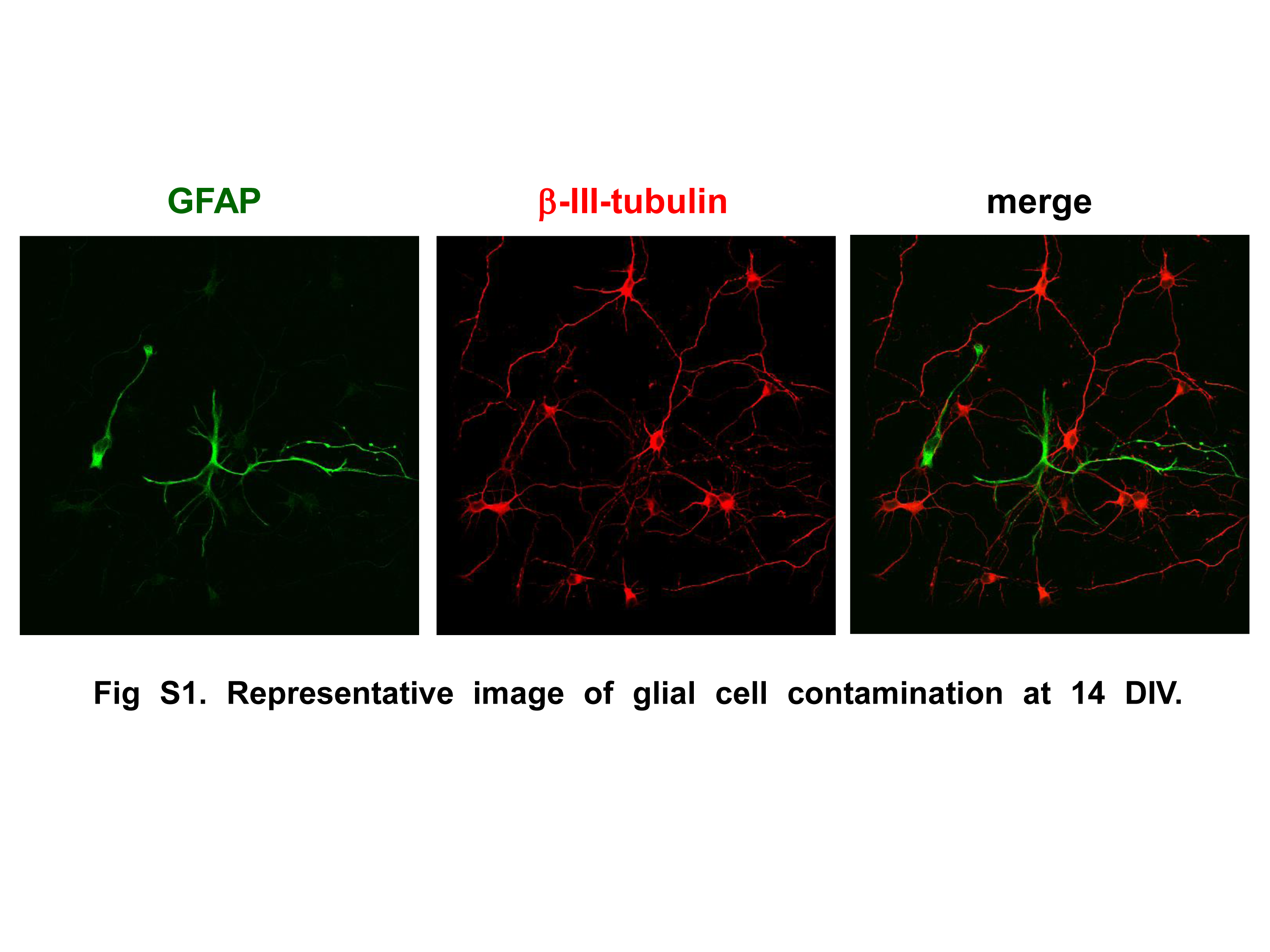

Supplement: Figure S1 — Representative image of glial cell contamination at 14 DIV. The percentage of glial contamination was obtained by counting the number of GFAP positive cells (green). Neurons are detected by b-III-tubulin immunostaining (red). (TIF) [file pone.0057626.s001.tif]

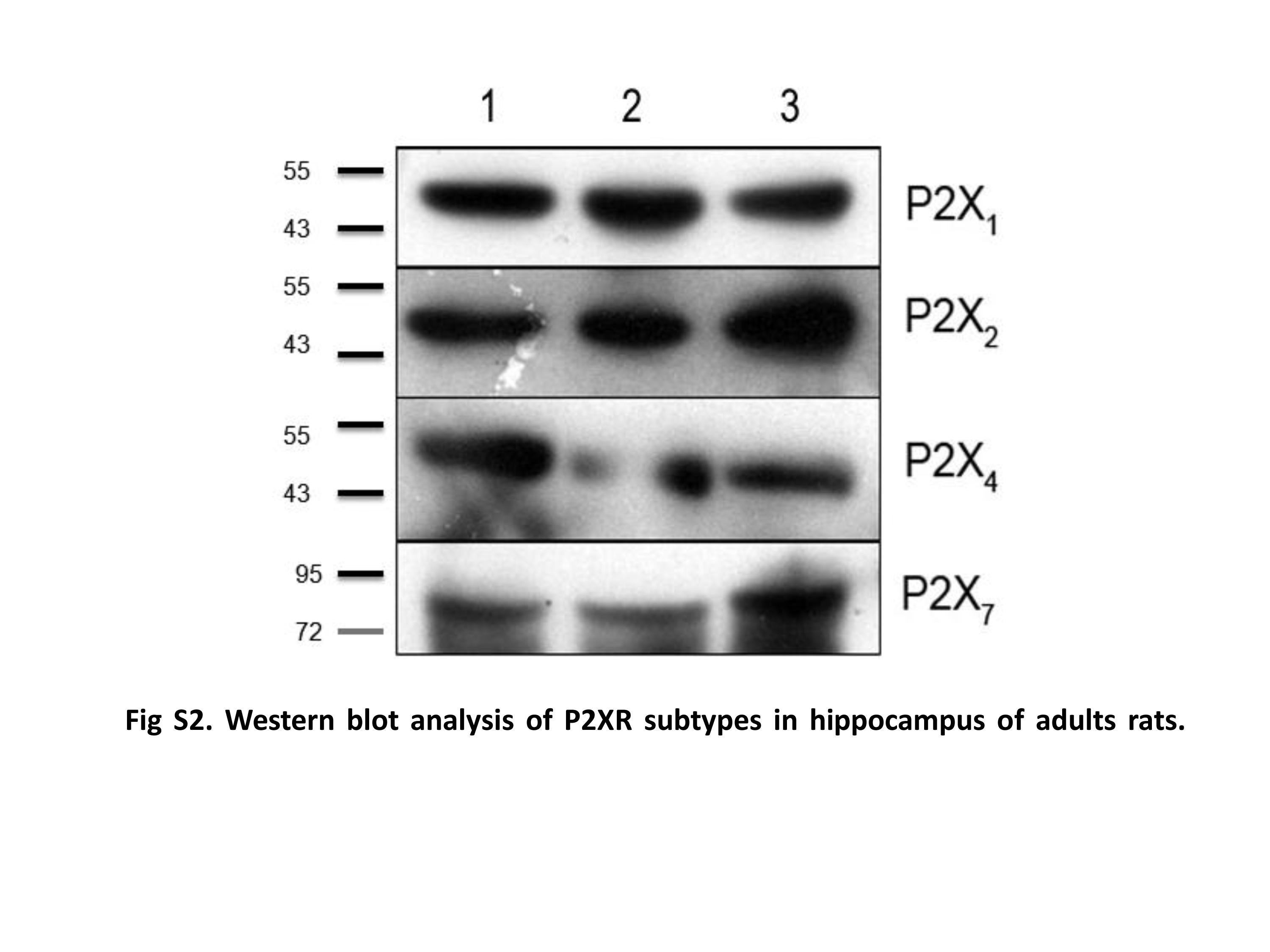

Supplement: Figure S2 — Western blot analysis of P2XR subtypes in hippocampus of adults rats. The fig. show the detection of P2X1, 2, 4 and 7 receptors, in three different rats. Bars indicate molecular weights in KD. (TIF) [file pone.0057626.s002.tif]
